# Editorial: Recent advances in understanding the role and mechanisms of gut microbiota in inflammatory bowel disease and irritable bowel syndrome

**DOI:** 10.3389/fcimb.2024.1521676

**Published:** 2024-11-27

**Authors:** Abbas Yadegar, Aryan Salahi-Niri, Cong Dai, Jun Shen

**Affiliations:** ^1^ Foodborne and Waterborne Diseases Research Center, Research Institute for Gastroenterology and Liver Diseases, Shahid Beheshti University of Medical Sciences, Tehran, Iran; ^2^ Department of Gastroenterology, First Affiliated Hospital, China Medical University, Shenyang, Liaoning, China; ^3^ Renji Hospital, School of Medicine, Shanghai Jiao Tong University, Shanghai, China

**Keywords:** gut microbiota, inflammatory bowel disease (IBD), irritable bowel syndrome (IBS), dysbiosis, Crohn’s disease, ulcerative colitis

Crohn’s disease (CD) and ulcerative colitis (UC), collectively known as inflammatory bowel diseases (IBD), are chronic, relapsing inflammatory disorders of the gastrointestinal (GI) tract. IBD affects over 1 million individuals in the United States and approximately 2.5 million in Europe (Rubin et al., 2012; Hammer and Langholz, 2020). Although IBD is a global health concern with the highest prevalence in Western countries, newly industrialized regions in Asia, the Middle East, Africa, and South America have seen a rapid rise in the disease incidence (Hammer and Langholz, 2020). Despite extensive research, the precise etiology and pathogenesis of IBD remain unclear. However, genome-wide association studies have identified more than 200 IBD-associated susceptibility genes, several of which are involved in mediating host responses to the gut microbiota (Nishida et al., 2018; El Hadad et al., 2024). This finding has led to increasing interest in the role of the gut microbiota in IBD pathogenesis.

The human gut is the home to approximately 100 trillion microorganisms, including bacteria, viruses, fungi, and protozoa, collectively referred to as the gut microbiota. Using culture-independent molecular methods, it is estimated that over 1,000 species of bacteria inhabit the GI tract, and the combined genome of these intestinal microbes contains roughly 100 times more genes than the human genome (Alshehri et al., 2021). More than 99% of intestinal bacteria fall within four major phyla: Firmicutes, Bacteroidetes, Proteobacteria, and Actinobacteria. Of these, the two dominant phyla in healthy adults are Firmicutes and Bacteroidetes (Yuan et al., 2023). The abundance and composition of bacteria vary significantly across different regions of the GI tract. The stomach and upper small intestine host relatively few microbial species, while the number of bacteria increases progressively from the jejunum to the colon (El Hadad et al., 2024).

Li et al. employed a Mendelian randomization approach to explore the causal relationships between gut microbiota composition and subtypes of CD and UC. The researchers identified specific bacterial taxa, such as *Hungatella* and Acidaminococcaceae, as protective factors against certain subtypes of IBD, while others like *Terrisporobacter* and *Anaerostipes* were associated with increased risk. These findings suggested that dysbiosis may vary between IBD subtypes, with implications for personalized therapeutic interventions targeting specific microbial communities depending on the IBD subtype (Nishida et al., 2018).

Expanding on this, Kulecka et al. examined the interaction between gut microbiota and *Clostridioides difficile* infection (CDI) in patients with IBD and cancer-related diarrhea. The researchers found that IBD and cancer patients both exhibited gut dysbiosis characterized by reduced microbial diversity. CDI further exacerbated this dysbiosis, leading to a significant reduction in beneficial bacteria such as *Bacteroides fragilis*, while increasing the abundance of pathogenic species like *Escherichia coli* and *Klebsiella pneumoniae*. The reduction in short-chain fatty acid (SCFA)-producing bacteria (such as those generating butyrate and propionate) was linked to worsened gut inflammation and disruption of intestinal homeostasis (Quaglio et al., 2022).

Further exploring systemic connections, Du et al. investigated the concept of lung-gut crosstalk, highlighting the mutual influence between respiratory diseases and IBD. The authors suggested that both lungs and intestines are integral parts of the common mucosal immune system, with immune responses in one organ affecting the other. The review emphasized that gut microbial imbalances could contribute to respiratory diseases such as asthma and chronic obstructive pulmonary disease (COPD), while respiratory diseases could exacerbate IBD symptoms. The presence of dysbiosis in both conditions suggests potential for shared therapeutic strategies targeting gut microbiota to improve outcomes in both lung and intestinal diseases (Singha et al., 2023; Li et al., 2024).

In a different vein, Hou et al. focused on the role of the aryl hydrocarbon receptor (AHR) in the context of IBD, with particular attention to how gut microbiota influences AHR activation. The authors discussed how microbial metabolites, such as tryptophan derivatives, can activate AHR, which in turn plays a crucial role in regulating immune responses, inflammation, and maintaining gut barrier integrity. In IBD, a decrease in AHR activity was associated with more severe inflammation and dysbiosis, indicating that therapeutic strategies aimed at enhancing AHR activation could alleviate IBD symptoms. The review also highlighted the potential for non-toxic natural AHR ligands to present as new therapeutic agents.

In conclusion, the published studies in this Research Topic highlight the significant importance of gut microbiota in the pathogenesis and management of IBD and irritable bowel syndrome (IBS). Dysbiosis, or the disruption of microbial balance, is a common feature in both IBD and IBS, contributing to inflammation and disease progression (Nishida et al., 2018). Studies such as those by Kulecka et al. demonstrate how factors like CDI further exacerbate gut dysbiosis in IBD, while others, like Hou et al., emphasize the critical role of the AHR in regulating immune responses and maintaining gut barrier integrity. Additionally, the concept of lung-gut crosstalk discussed by Du et al. underscores the systemic nature of gut health, suggesting that targeting microbiota may benefit extraintestinal conditions as well. Finally, the findings of Li et al. reveal how microbiota composition varies across IBD subtypes, supporting the potential for personalized therapeutic strategies. Collectively, these studies reinforce the pivotal role of the gut microbiota in IBD and IBS and point toward promising microbiota-targeted therapies as well as personalized approaches for improving patient outcomes (Quaglio et al., 2022).
